# An implementation research agenda

**DOI:** 10.1186/1748-5908-4-18

**Published:** 2009-04-07

**Authors:** Martin P Eccles, David Armstrong, Richard Baker, Kevin Cleary, Huw Davies, Stephen Davies, Paul Glasziou, Irene Ilott, Ann-Louise Kinmonth, Gillian Leng, Stuart Logan, Theresa Marteau, Susan Michie, Hugh Rogers, Jo Rycroft-Malone, Bonnie Sibbald

**Affiliations:** 1 Institute of Health and Society, Newcastle University, Newcastle upon Tyne, UK; 2 Division of Health and Social Care Research, Kings College, London, UK; 3 Department of Health Sciences, University of Leicester, Leicester, UK; 4 National Patient Safety Agency, London, UK; 5 School of Management, University of St Andrews, St Andrews, UK; 6 National Institute for Health Research Service Delivery and Organisation Programme, London School of Hygiene and Tropical Medicine, London, UK; 7 Centre for Evidence-Based Medicine, Department of Primary Health Care, University of Oxford, Oxford, UK; 8 Institute of Work Psychology, University of Sheffield, Sheffield, UK; 9 General Practice and Primary Care Research Unit, University of Cambridge, Cambridge, UK; 10 National Institute for Health and Clinical Excellence, London, UK; 11 NIHR PenCLAHRC, Peninsula College of Medicine and Dentistry, Universities of Exeter and Plymouth, UK; 12 Institute of Psychiatry, Kings College, London, UK; 13 Centre for Outcomes Research and Effectiveness, Department of Psychology, University College London, London, UK; 14 Service Transformation, NHS Institute for Innovation and Improvement, Coventry House, University of Warwick Campus, Coventry, UK; 15 School of Healthcare Sciences, Bangor University, Bangor, UK; 16 National Primary Care Research and Development Centre, University of Manchester, Manchester, UK

## Abstract

In October 2006, the Chief Medical Officer (CMO) of England asked Professor Sir John Tooke to chair a High Level Group on Clinical Effectiveness in response to the chapter 'Waste not, want not' in the CMOs 2005 annual report 'On the State of the Public Health'. The high level group made recommendations to the CMO to address possible ways forward to improve clinical effectiveness in the UK National Health Service (NHS) and promote clinical engagement to deliver this. The report contained a short section on research needs that emerged from the process of writing the report, but in order to more fully identify the relevant research agenda Professor Sir John Tooke asked Professor Martin Eccles to convene an expert group – the Clinical Effectiveness Research Agenda Group (CERAG) – to define the research agenda. The CERAG's terms of reference were 'to further elaborate the research agenda in relation to pursuing clinically effective practice within the (UK) National Health Service'. This editorial presents the summary of the CERAG report and recommendations.

## Background

In October 2006, the Chief Medical Officer (CMO) of England asked Professor Sir John Tooke to chair a High Level Group on Clinical Effectiveness in response to the chapter 'Waste not, want not' in the CMOs 2005 annual report 'On the State of the Public Health'. The High level group made recommendations to the CMO to address possible ways forward to improve clinical effectiveness in the UK National Health Service (NHS) and promote clinical engagement to deliver this. The report contained a short section on research needs that emerged from the process of writing the report, but in order to more fully identify the relevant research agenda Professor Sir John Tooke asked Professor Martin Eccles to convene an expert group – the Clinical Effectiveness Research Agenda Group (CERAG) – to define the research agenda. The CERAG's terms of reference were 'to further elaborate the research agenda in relation to pursuing clinically effective practice within the (UK) National Health Service'.

Terminology is a problem in both the practice of, and researching into, clinical effectiveness. The high level group uses the term 'clinical effectiveness' as it built on the terminology used within the CMO's report. However, a study of 33 applied research funding agencies across nine countries identified 29 terms used to refer to some aspect of the processes around clinically effective practice [1]. This confusion has been compounded by the recent prominence of 'Translational Research', and the description of the first and second translation gaps. Given the balance of scientific endeavour and funding, the term 'Translational Research' is mainly thought of as the T1 bench to bedside process of transferring basic science knowledge into new drugs and technologies. Attracting about 1% of the research funding devoted to T1 research the T2 Translational Research is the process of taking current scientific knowledge and ensuring it is applied in routine clinical care [2].

Within the UK, the terms 'Implementation' and 'Implementation Research' seem to be the best recognised. Therefore, as a focus for its deliberations the CERAG adopted the following definition:

'Implementation Research is the scientific study of methods to promote the systematic uptake of clinical research findings and other evidence-based practices into routine practice, and hence to improve the quality (effectiveness, reliability, safety, appropriateness, equity, efficiency) of health care. It includes the study of influences on healthcare professional and organisational behaviour.' (adapted from Implementation Science  accessed 10 February 2009).

This editorial presents the summary of the group's report and recommendations; the full report is available as Additional File 1.

## The importance of Implementation Research and its funding

The findings from clinical and health services research can not change population health outcomes unless health care systems, organizations, and professionals adopt them in practice [3]. A consistent finding is that the transfer of research findings into practice is unpredictable and can be a slow and haphazard process. The relative inattention to implementing what we know is costing lives. There is an imbalance between investment in the development of new drugs and technologies versus improving the fidelity with which care is delivered.

In a structured review of healthcare professionals views on clinician engagement in quality improvement, Davies *et al*. identified 86 empirical reports relevant to the review [4]. They report that the literature suggests: healthcare professionals are heterogeneous in relation to their definition of quality; their perception of the need for quality improvement; their attitudes to quality improvement initiatives; their attitudes to clinical guidelines and evidence-based practice. In addition, they have a limited understanding of the concepts and methods of quality improvement, and quality improvement is often the scene of turf battles. Under the heading of perceived barriers, they also stated that 'many of the identified barriers arise from the well-documented problems of working effectively between and across health professions. This means that although more time and more resources may be necessary or helpful (directly and in their explicit recognition of healthcare professionals' concerns), they are unlikely to be sufficient on their own to overcome the substantial barriers to clinicians' active engagement in successful quality improvement'. Healthcare professionals are an important part of the organisation in which they work (and are subject to organisational policies, procedures, and cultures); this review offers a partial explanation for the persistent quality gaps and also supports the contention that it is unlikely that this will change spontaneously.

Recognition of quality gaps has led to increased interest in more active implementation strategies. Over the past 10 years, a body of Implementation Research has developed [5-7]. This demonstrates that interventions can be effective, but provides less information to guide the choice or optimise the components of such complex interventions in practice [8]. While the effectiveness of interventions varies across different clinical problems, contexts, and organizations, studies provide scant theoretical or conceptual rationale for their choice of intervention [9], and only limited descriptions of the interventions and contextual data [6]. Research on economic and political approaches to change is scarce [10], and it is therefore not surprising that little is known about how best to integrate disease and case management interventions into existing healthcare at the system level. Thus, the science of Implementation Research is still a work in progress, largely due to the fact that it is a relatively young science.

Internationally, Implementation Research is a recognised area of funding within other healthcare systems; this is not the case in the UK. The Cooksey Report [11] suggested a UK annual research budget (Public sector and major charities) of just over £2 billion. The proportion spent on health services (as opposed to biomedical or clinical) research in general is small. While there have been a number of previous funding programmes for Implementation Research within the UK, none are current. The proportion of annual research money devoted to Implementation Research is impossible to quantify; it is likely to be of the order of a maximum of a few millions pounds per year.

The Cooksey Report [11], having identified the need for implementation and Implementation Research, offers a sound basis on which to elaborate the Implementation Research agenda as a core part of a research agenda of key relevance to the NHS.

One of the major problems with not having a clearly identified, named Implementation Research funding stream is that the whole area loses 'profile'; the issues become blurred and the central focus of the routine uptake of findings, from clinical research programmes into routine care, becomes lost to research enquiry. In countries where there is a named, dedicated, funding stream (*e.g*., Canada, Australia) the research area has a higher profile with both researchers and with clinicians. There is the potential for senior researchers to establish programmes of research (rather than doing one-off studies), junior researchers to make it a career choice, and clinicians to become willing collaborators, thereby facilitating the spread of knowledge and the improvement of methods.

## Specific considerations for an Implementation Research agenda

In elaborating the Implementation Research agenda the, CERAG identified five important overarching considerations that should influence thinking about, and commissioning of, Implementation Research.

First, it is important to consider the multiple levels at which healthcare is delivered and the interplay between them in their cultural context [12].

Second, Implementation Research centrally involves the study of changing behaviour and maintaining change – in organizations, and the groups and individual healthcare professionals within them.

Third, the use of theory in Implementation Research offers (at least) three important potential advantages. Theories offer a generalisable framework that can apply across differing settings and individuals; they offer the opportunity for the incremental accumulation of knowledge; and they offer an explicit framework for analysis. The CERAG agreed that appropriate consideration of theory was an important element of Implementation Research. As well as a more thoughtful use of theory, there is a need to work through the various stages of using theory and resolving such apparently simple issues as what it means for an intervention to be theory-based or what is the theoretical basis of behaviour change.

Fourth, frameworks are potentially useful tools for considering the issues that a research agenda needs to address [13]. Inevitably there is no one ideal, universally accepted framework that will fit all purposes; different frameworks will often reflect different purposes, disciplinary, or philosophical standpoints, and so will appeal to different groups or individuals.

Fifth, a general complaint of implementation studies (often trials) is that the need for experimental control, maximising internal validity, compromises external validity. As ever, the balance of considering these two dimensions of validity depends on the question that is being answered at the time [14].

## Who is this research agenda aimed at?

This discussion of the research agenda is aimed primarily at commissioners of research, but will also be of relevance to a broader range of policy makers and researchers. While this report has been discussed and written in the context of the UK National Health Service and the National Institute for Health Research (NIHR) it is possible that a variety of other research-commissioning organisations could use it to identify areas that are a priority for them. However, it has been considered in its entirety and, in terms of programmatic commissioning, a piecemeal approach to addressing it could leave important areas unaddressed.

## A Research Agenda

### Research areas

Many of these research areas are interlinked. The CERAG offered exemplar questions within each of them in order to illustrate key issues. The processes suggested in the subsequent recommendations will further elaborate and prioritise the content of this agenda.

### Context

The impact of context on implementation is important, and systematic study of the attributes of context (and their role and modifiability) that form barriers or facilitators to implementation is needed. The responsiveness of context is important in order to understand (and influence) culture and other attributes of organisations as well as the individuals within them and their interests related to implementation of new knowledge. The role of context in intervention development needs to be better understood.

### Behavioural determinants and evaluation of change strategies

Successful implementation of new knowledge should be built on an understanding of the determinants of behavioural change and maintenance of behavioural change in individuals and organisations. Such understanding would allow the rational development and testing of implementation interventions. This should include the systematic development of interventions and trials across a range of conditions and NHS settings. These could include the study of the organisational embedding of new interventions, the effectiveness of healthcare system interventions, as well as evaluation of delivering new models and methods of care. There is a need for studies examining the methods of optimising the content and methods of delivery of interventions.

Evaluations should use a range of (and often a combination of) research designs and methods (*e.g*., cluster randomized trials, quasi-experimental designs, and qualitative studies).

### Testing of theory in Implementation Research

Theory is underused in Implementation Research. There needs to be considerable work on understanding available theories, on the testing and development of theories, and on how to operationalise theory. This work should not be restricted by disciplinary perspectives, worldview, or area of application.

### Knowledge attributes and knowledge generation – features related to uptake

Research is needed on the important attributes of new knowledge and how these influence its uptake (or not). This would include the attributes of and applicability of what is regarded as evidence by different individuals and in different contexts.

Decision makers have problems accessing, appraising, adapting, and applying research evidence. The increasing recognition that implementation of evidence from individual studies may be misleading, either due to bias in their conduct or random variations in findings, has led to greater emphasis on knowledge syntheses as the basic unit of implementation. Knowledge syntheses interpret the results of individual studies within the context of global evidence thus increasing the 'signal to noise ratio' of implementation activities and increasing the likelihood of their success. Knowledge syntheses provide the evidence base for other implementation vehicles such as patient decision aids, clinical practice guidelines, or policy briefs.

Systematic review activities (guided by relevant theory) need to be supported systematically to ensure their continued development. Important areas activity include: compiling and maintaining a register of systematic reviews of Implementation Research; updating overviews of reviews of professional behaviour change interventions; conducting systematic reviews of methods to improve the implementation of clinical research findings in routine settings; workshops on conduct and use of knowledge syntheses targeted to different stakeholders.

### Cross-cutting issues

#### Methodology

Across all of the areas above there will be important methodological issues that need to be identified, investigated, and resolved. These include:

1. The area of Implementation Research needs a common understanding of terms. Important areas of research include: the development of one or more taxonomies of barriers to implementation, mediating mechanisms and pathways; standardised measurement approaches for key elements of the taxonomy; a suite of reporting guidelines for different types of Implementation Research.

2. All of the areas pose measurement challenges, such as the development of process and outcome methods and measures for relevant constructs.

3. Is there a 'core set' of measures that will be applicable to most settings, or is each combination of patient team and organisation conceptually unique? The idea of a core set of measures offers greater potential for accumulation of knowledge.

4. What are the pros and cons of using proxies for behaviour, such as written or web-based vignettes that simulate clinical behaviours?

5. The incorporation of economic analysis within Implementation Research is not necessarily methodologically challenging, but it is very uncommon and should be encouraged and supported.

6. An explicit examination of the pros and cons of the use of routinely available data to assess implementation. This would include the availability of data and the specificity of data in relation to the implementation of research evidence. Are there situations where there is sufficient routinely available data for economic modelling to demonstrate the viability or otherwise of certain behaviour change strategies? How complex can and should such modelling become?

#### Implementation Research across different areas of clinical practice

Implementation Research will be conducted in a range of clinical areas. This needs to be done in a way that ensures contribution to an incremental understanding of implementation. Research in one clinical area should generate ideas and understanding that can be drawn on in other clinical areas.

#### Knowledge infrastructure for Implementation

This links to 'knowledge attributes', (above) and is addressed in the UK by initiatives such as the NHS National Library for Health, the Cochrane Collaboration and Social Care Online. Nonetheless, the process recommended below could formally set out the knowledge infrastructure for implementation. This would be an important exercise in making explicit the content of an infrastructure (staff, skills, and resources), its scale, and its degree of current (and future) integration into routine healthcare.

#### Sustainability

The consideration of sustainability permeates the research agenda. It is important to have a healthcare workforce that can sustain implementation in the clinical setting as a matter of routine. It is important that we learn more about the organisational/contextual factors that enable the sustained use of evidence in practice. It is also important to have a research workforce that can sustain the area of Implementation Research.

Within research itself it is important to examine attributes of sustainability (within individuals, teams, and organizations) and to develop methods to examine whether the effects of interventions are sustained over time.

#### Communication strategy/engagement with the NHS

As part of integrating implementation and Implementation Research within the NHS it will be vital to develop an explicit communication and engagement strategy.

### Workforce issues

#### Capacity to do implementation

How should the NHS workforce (clinicians/practitioners and managers) be trained (at both undergraduate and postgraduate levels) in order to optimise their ability to implement new knowledge (without doing harm, overspending, giving more to one patient than another, while also stopping ineffective practices)?

What are effective engagement strategies to involve the workforce in implementation?

What are the important attributes of the workforce that enhance knowledge use and implementation in healthcare settings?

How can these attributes be sustained both within individuals and organisations?

#### Capacity to do Implementation Research

Capacity to do research into implementation is limited both within the UK and internationally. The NIHR needs a strategy of building capacity at all levels of the researcher career. Given the time that it takes to build experience in this area NIHR needs a cadre of experienced senior investigators who can direct programmes of research.

A funding strategy should also train junior researchers to be capable of developing into independent researchers (this should be linked with experience Implementation Researchers). This could involve a mix of PhD studentships and fellowship awards.

#### Attributes of research teams addressing this agenda

Addressing this research agenda will be an inherently multi- and inter-disciplinary endeavor. No one practice or academic group or discipline will bring all the necessary attributes to address the research agenda. The range of required disciplines will vary within and across the various areas of the research agenda, but is likely to include some of Implementation Research, sociology, health psychology, health economics, and statistics.

### Implementation and evidence of benefit from clinical and public health interventions

It will most often be the case that the Implementation Research agenda will be applied to areas where there is a clear understanding of appropriate clinical care or public health practice. In some areas there will be insufficient published evidence to inform a clear, shared understanding of optimum practice; in such instances the research agenda should address the need for evidence of efficient clinical and public health practice.

## Recommendations

1. NIHR should initiate a process to establish a research programme within NIHR with an explicit dedicated, protected, funding stream for funding Implementation Research.

a. This process should detail issues such as:

i. the scope and prioritization of topics for such a programme.

ii. the potential overlap with current national research programmes within and outwith NIHR.

iii. the potential overlap with other NIHR funded initiatives – National Library for Health, Collaborations for Leadership in Applied Health Research and Care (CLAHRCs), Cochrane Collaboration.

iv. the relevant stakeholders in the process.

v. the appropriate configuration of such a programme of research – either as a single entity (maximising focus, scarce researcher resources, and critical mass), or as a dimension of each of the current national programmes (more diffuse, but probably more administratively straightforward to establish).

vi. the establishment of a commissioning group with appropriate expertise to evaluate proposals.

vii. the timescale for establishing launching and commissioning research within such a programme.

viii. relevant indicators of success for such a programme to allow its evaluation.

b. Given the scale for return on investment and potential to save lives, this should aim to achieve a steady-state annual budget of 2 to 3% of NIHR total research budget. With total budget estimates at £750 million, this equates to approximately £15 to 22 million.

c. Spending on this scale will not be achievable immediately, and so the process should consider an escalating funding process starting at a lower level and incrementally rising to the steady-state figure over a number of years.

d. Long-term commitment is needed to deal with the issue of creating a climate conducive to conducting Implementation Research and the closely linked area of using research findings in routine settings. Without this being seen as both central and important, it is unlikely to be sustained.

e. Consideration should be given to the idea of establishing one or more Centers of Implementation Research Excellence along the lines of the Public Health Centers of Excellence.

2. A mix of project and programme funding would allow studies of a shorter and more 'worked through' nature, as well as series of interlinked conceptual, methodological work that is needed in the area.

3. The process of commissioning should be a mix of commissioner-defined and curiosity-driven. In such a relatively young area, it is unlikely to be possible for a commissioned research process to fully cover all relevant areas, particularly in the areas of methodological and conceptual work.

4. In order to enhance capacity development, a proportion of the funding should be directed towards studentships, fellowships, and bursaries.

5. There should be consideration of the development of training programmes for Implementation Researchers. Although not a research budget cost, there should also be consideration of the development of (pre- and post-registration) training programmes for clinicians and non-clinicians within the NHS around building capacity to better use implementation (and clinical) research in daily practice.

6. Implementation Research and Implementation Researchers need to be embedded within the NHS. One way to achieve this would be to consider further strengthening and extending the Implementation Research dimensions of the Collaboration in Applied Health Research and Care centers. This should also consider how to closely ally those researching implementation with those doing implementation on a daily basis.

7. In order to advance the research area, funding should be directed towards providing opportunities for scientists and clinicians to meet to discuss relevant issues – akin to the UK Economic and Social Research Council Seminar Series Grants.

8. NIHR should give consideration to establishing a standing advisory group, with appropriate expertise, to continue to develop, oversee, and advise on Implementation Research within the NHS. Such a body could also make links with other national centers to form an international network.

## Competing interests

The CERAG members are researchers, policy makers, or research funders in areas in some way allied to Implementation Research.

## Authors' contributions

MPE convened and chaired the CERAG. All group members contributed to the content of the report through either face to face meeting or comment on sequential drafts. MPE drafted the report and this article. All Group members agreed the submitted version of the report and this article.

## Supplementary Material

Additional File 1 **CERAG Report**. A report prepared for the High Level Group on Clinical Effectiveness by the Clinical Effectiveness Research Agenda Group. Click here for file
