# Supplementary material for: An implementation research agenda
Source: Implement Sci. 2009 Apr 7;4:18. doi: 10.1186/1748-5908-4-18 (PMC2671479; doi:10.1186/1748-5908-4-18)
Supplement: Additional File 1 — CERAG Report. A report prepared for the High Level Group on Clinical Effectiveness by the Clinical Effectiveness Research Agenda Group. [file 1748-5908-4-18-S1.pdf]

An Implementation Research Agenda

A report prepared for the High Level Group on Clinical Effectiveness

by

The Clinical Effectiveness Research Agenda Group

Contents

1. Executive Summary & Recommendations ... 3
2. Background, Terms of Reference and Implementation research ... 7
    2.1. Introduction ... 7
    2.2. Terms of reference of CERAG ... 9
    2.3. Defining evidence-based clinical effectiveness ... 9
    2.4. Research into clinical effectiveness ... 10
3. The importance of Implementation Research and its funding ... 11
    3.1. Why is Implementation Research important? ... 11
    3.2. Patterns of research funding in the UK ... 16
    3.3. Previous funding for Implementation Research ... 16
    3.4. Implementation research funding internationally ... 20
    3.5. The profile of Implementation Research within NIHR ... 21
4. Specific considerations for an Implementation Research Agenda ... 22
    4.1. Levels of engagement - seeing the whole picture ... 22
    4.2. Behaviour change ... 23
    4.3. Theories, Frameworks and Models ... 23
    4.4. Balancing internal and external validity ... 26
5. The research agenda ... 28
    5.1. Who is this research agenda aimed at? ... 28
    5.2. Research areas ... 29
    5.3. Cross cutting issues ... 30
    5.4. Workforce issues ... 31
    5.5. Attributes of research teams addressing this agenda ... 32
    5.6. Implementation and evidence of benefit from clinical and public health interventions ... 32
6. Recommendations ... 33

Appendix 1. Membership of the Clinical Effectiveness Research Agenda Group ... 35

Appendix 2 Selected sections from the Cooksey Report relating to the second gap in translation ... 36

1. Executive Summary & Recommendations

Chapter 1 Executive Summary

Chapter 2 Background, Terms of Reference and Implementation research

In October 2006 the Chief Medical Officer asked Professor Sir John Tooke to chair a High Level Group on Clinical Effectiveness in response to the chapter "Waste not, want not" in his 2005 report *On the State of the Public Health*.

Following the submission of the report, in order to more fully identify the relevant research agenda Professor Sir John Tooke asked Professor Eccles to convene an expert group – the Clinical Effectiveness Research Agenda Group (CERAG) - to define the research agenda.

The CERAG's terms of reference are 'to further elaborate the research agenda in relation to pursuing clinically effective practice within the NHS'.

Within the UK the terms "Implementation" and "Implementation Research" seem to be the best recognised. Therefore, as a focus for its deliberations the CERAG adopted the following definition: "Implementation research is the scientific study of methods to promote the systematic uptake of clinical research findings and other evidence-based practices into routine practice, and hence to improve the quality (effectiveness, reliability, safety, appropriateness, equity, efficiency) of health care. It includes the study of influences on healthcare professional and organisational behaviour."

Chapter 3 The importance of Implementation Research and its funding

The findings from clinical and health services research can not change population health outcomes unless health care systems, organizations and professionals adopt them in practice. A consistent finding is that the transfer of research findings into practice is unpredictable and can be a slow and haphazard process.

The relative inattention to implementing what we know is costing lives. There is an imbalance between investment in the development of new drugs and technologies versus improving the fidelity with which care is delivered.

The Cooksey Report suggested an annual research spend (Public sector and major charity) of just over £2 billion. The proportion spent on Health Services (as opposed to biomedical or clinical) Research in general is small. The proportion spent on Implementation Research is impossible to quantify; it is likely to be of the order of a maximum of a few millions of pounds per year.

There have been a number of previous funding programmes for Implementation Research; none are current. The Cooksey Report, having identified the need for implementation and implementation research, offers a sound basis on which to elaborate the implementation research agenda as a core part of a research agenda of key relevance to the NHS.

Internationally, Implementation Research is a recognised area of funding within other healthcare systems.

One of the major problems with not having a clearly identified, named Implementation Research funding stream is that the whole area loses "profile"; the issues become blurred and the central focus of the routine uptake of findings from clinical research programmes into routine care becomes lost to research enquiry.

Chapter 4 Specific considerations for an Implementation Research agenda

In elaborating the Implementation Research agenda we identified a number of important overarching considerations that should influence thinking about and commissioning Implementation Research.

It is important to consider the multiple levels at which healthcare is delivered and the interplay between them in their cultural context.

Implementation research centrally involves the study of changing behaviour and maintaining change – in organizations and the groups and individual healthcare professionals within them.

The use of theory in Implementation Research offers (at least) three important potential advantages. Theories offer a generalisable framework that can apply across differing settings and individuals; they offer the opportunity for the incremental accumulation of knowledge; and they offer an explicit framework for analysis.

Frameworks are potentially useful tools for considering the issues that a research agenda needs to address. Inevitably there is no one ideal, universally accepted framework that will fit all purposes; different frameworks will often reflect different purposes, disciplinary or philosophical standpoints and so will appeal to different groups or individuals.

A general complaint of implementation studies (often trials) is that the need for experimental control, maximising internal validity, compromises external validity. As ever, the balance of considering these two dimensions of validity depends on the question that is being answered at the time.

Chapter 5 The Research Agenda

This discussion of the research agenda is aimed primarily at commissioners of research but will also be of relevance to a broader range of policy makers and researchers.

Many of the research areas are interlinked. Within the chapter we offer exemplar questions within each of them in order to illustrate key issues.

- The research areas are:
    - Context; Behavioural determinants and evaluation of change strategies; Testing of theory in implementation research; Knowledge attributes and knowledge generation

- Cross cutting issues
    - Methodology; Implementation Research across different areas of clinical practice; Knowledge infrastructure for Implementation; Sustainability
- Communication strategy/engagement with the NHS
- Workforce issues
  - Capacity to do implementation; Capacity to do implementation research
- Attributes of research teams addressing this agenda
- Implementation and evidence of benefit from clinical and public health interventions

Chapter 6 Recommendations

1. NIHR should initiate a process to establish a research programme within NIHR with an explicit dedicated, ring-fenced, funding stream for funding Implementation Research.
    a. This process should detail issues such as:
        i. the scope and prioritization of topics for such a programme;
        ii. the potential overlap with current national research programmes within and outwith NIHR;
        iii. the potential overlap with other NIHR funded initiatives – NELH, CLAHRCS, Cochrane Collaboration
        iv. the relevant stakeholders in the process;
        v. the appropriate configuration of such a programme of research – either as a single entity (maximising focus, scare researcher resources and critical mass) or as a dimension of each of the current national programmes (more diffuse but probably more administratively straight forward to establish)
        vi. the establishment of a commissioning group with appropriate expertise to evaluate proposals;
        vii. the timescale for establishing launching and commissioning research within such a programme;
        viii. relevant indicators of success for such a programme to allow its evaluation.
    b. Given the scale for return on investment and potential to save lives this should aim to achieve a steady state annual spend of 2-3% of NIHR total research spend. With total spend estimated at £750 million this equates to approximately £15 - 22 million.

c. Spending on this scale will not be achievable immediately and so the process should consider an escalating funding process starting at a lower level and incrementally rising to the steady state figure over a number of years.

d. Long term commitment is needed to deal with the issue of creating a climate conducive to conducting implementation research and the closely linked area of using research findings in routine settings. Without this being seen as both central and important it is unlikely to be sustained.

e. Consideration should be given to the idea of establishing one or more Centres of Implementation research Excellence along the lines of the Public Health Centres of Excellence.

2. A mix of Project and Programme funding would allow studies of a shorter and more “worked through” nature as well as series of interlinked conceptual, methodological work that is needed in the area.

3. The process of commissioning should be a mix of commissioner defined and curiosity driven. In such a relatively young area it is unlikely to be possible for a commissioned research process to fully cover all relevant areas, particularly in the areas of methodological and conceptual work.

4. In order to enhance capacity development a proportion of the funding should be directed towards studentships, fellowships and bursaries.

5. There should be consideration of the development of training programmes for implementation researchers. Although not a research budget cost there should also be consideration of the development of (pre and post-registration) training programmes for clinicians and non-clinicians within the NHS around building capacity to better use implementation (and clinical) research in daily practice.

6. Implementation research and implementation researchers need to be embedded within the NHS. One way to achieve this would be to consider further strengthening and extending the implementation research dimensions of the CLAHRCS centers. This should also consider how to closely ally those researching implementation with those doing implementation on a daily basis.

7. In order to promote advancement of the research area funding should be directed towards providing opportunities for scientists and clinicians to meet to discuss relevant issues – akin to the ESRC Seminar Series Grants.

8. NIHR should give consideration to establishing a standing advisory group, with appropriate expertise, to continue to develop, oversee, and advise on implementation research within the NHS. Such a body could also make links with other national centres to form an international network.

2. Background, Terms of Reference and Implementation research

Chapter Summary

In October 2006 the Chief Medical Officer asked Professor Sir John Tooke to chair a High Level Group (HLG) on Clinical Effectiveness in response to the chapter "Waste not, want not" in his 2005 report *On the State of the Public Health*.

Following the submission of the report, in order to more fully identify the relevant research agenda Professor Sir John Tooke asked Professor Eccles to convene an expert group – the Clinical Effectiveness Research Agenda Group (CERAG) - to define the research agenda.

The CERAG's terms of reference are 'to further elaborate the research agenda in relation to pursuing clinically effective practice within the NHS'.

Within the UK the terms "Implementation" and "Implementation Research" seem to be the best recognised. Therefore, as a focus for its deliberations the CERAG adopted the following definition: "Implementation research is the scientific study of methods to promote the systematic uptake of clinical research findings and other evidence-based practices into routine practice, and hence to improve the quality (effectiveness, reliability, safety, appropriateness, equity, efficiency) of health care. It includes the study of influences on healthcare professional and organisational behaviour."

2.1. Introduction

In October 2006 the Chief Medical Officer asked Professor Sir John Tooke to chair a High Level Group (HLG) on Clinical Effectiveness in response to the chapter "Waste not, want not" in his 2005 report *On the State of the Public Health*. The scope of the HLG's work was defined by the findings of this report:

- Variation in the provision of specific health services may be appropriate but it can also suggest waste or inequity within the National Health Service (NHS).
- Both under-use and over-use of medical interventions can be costly and expose patients to unnecessary risk.
- Variation that cannot be explained by the needs of patients may occur on the basis of geography ("postcode prescribing"), on account of the preferences and habits of clinical decision makers, or due to other factors, such as the socioeconomic status of patients.
- Variation is demonstrable in many areas of medical practice, including prescribing patterns, hysterectomy, treatment for people with coronary disease, and tonsillectomy among children.
- Inappropriate variation may be a function of poor knowledge, the flawed application of the correct knowledge, a lack of resources, or the inappropriate allocation of extant resources.

- The efforts of the National Institute for Health and Clinical Excellence (NICE) and NHS Connecting for Health in the effective dissemination of knowledge should be redoubled.
- Commissioners of health services should reaffirm their commitment to the NHS principle of equity, and techniques should be developed further to facilitate benchmarking of provision.
- NICE should be asked to issue guidance to the NHS on disinvestment, away from established interventions that are no longer appropriate or effective, or do not provide value for money.

The HLG's terms of reference were:

- To identify illustrative and informative examples of major clinical significance drawn from major domains of clinical care in England where there are significant variations in (evidence-based) best practice and/or where efficiency and organisation of care fall short of international benchmarks.
- To review the evidence base for measures aimed at enhancing the effectiveness and efficiency of clinical care that might be applied to these domains.
- Drawing on these analyses, to recommend a programme of action to enhance the effectiveness and efficiency of clinical care, including specific incentives that might be employed.

The HLG subsequently commissioned Professor Martin Eccles of the Institute of Health and Society at Newcastle University to summarise existing activity within government, the NHS, the Royal Colleges and professional and other relevant bodies, and set out current thinking on clinical effectiveness and barriers to implementation of guidelines and the boundaries of current knowledge. His work formed Part 2 of the HLG Report. It showed that there is a wide range of activities aimed at supporting clinical effectiveness ongoing within the NHS, but the degree to which they are fully integrated and aware of each other's activities is not clear. Additionally, it is not clear which organisation has lead responsibility for the several dimensions of clinical effectiveness. The report also identifies the rapidly increasing volume of evidence available to inform promotion of clinical effectiveness. Professor Eccles proposed that a series of explicit frameworks would be useful in order to address systematically the promotion of clinical effectiveness and suggested that such frameworks are not currently commonly used.

The HLG considered this evidence and identified specific underlying issues, and has made recommendations to the Chief Medical Officer to address them. These include possible ways forward to improve clinical effectiveness in the NHS and promote clinical engagement to deliver this. The report contained a short section on research needs that emerged from the process of writing the report but in order to more fully identify the relevant research agenda Professor Sir John Tooke asked Professor Eccles to convene an expert group – the Clinical Effectiveness Research Agenda Group (CERAG) - to define the research agenda.

2.2. Terms of reference of CERAG

The CERAG's terms of reference are 'to further elaborate the research agenda in relation to pursuing clinically effective practice within the NHS'. The deliberations of this group will form a report to the chair of the High Level Group, Professor Sir John Tooke. The members of CERAG are listed in Appendix 1.

2.3. Defining evidence-based clinical effectiveness

The HLG Report posed the question "What do we mean by evidence-based clinical effectiveness (EBCE)?" going on to state "What are the core activities that comprise EBCE, not only for clinicians but for all staff working in the NHS? There are current definitions of clinical effectiveness (e.g. the NHSE 1996 definition of clinical effectiveness and the domain outcome in DH *Standards for Better Health*)[1] but these do not automatically translate into the structure, staffing and skills that would be required. Defining these (by some means) would be a large step along the road to defining what a systematic approach to EBCE would look like.

Whilst this was an important consideration for the HLG the recent publications by NICE have gone some way towards providing both a framework for structures and for suggested activities within its "How to …" guides.[23]

**Box 1. Principles of implementation** (from How to put NICE guidance into practice: A guide to implementation for organisations [2)]<br><br>Evidence and experience shows that there are six key components to a successful implementation process:<br>• board support and clear leadership<br>• provision of a dedicated resource (a NICE manager)<br>• support from a multidisciplinary team<br>• a systematic approach to financial planning<br>• a systematic approach to implementing guidance<br>• a process to evaluate uptake and feedback.<br>The organisation's approach to meeting these components should be clearly stated in an implementation policy. This should be agreed across the organisation or health community and approved by the boards of the organisations involved.

---

1 The extent to which specific clinical interventions, when deployed in the field for a particular patient or population, do what they are intended to do – i.e. maintain and improve health and secure the greatest possible health gain from the available resources." Promoting Clinical Effectiveness – A framework for action in and through the NHS. NHSE, 1996.

"Patients achieve health benefits that meet their individual needs through health care decisions and services based on what assessed research evidence has shown provides effective clinical outcomes." In: Standards for Better Health. Department of Health, July 2004.

2 How to put NICE guidance into practice: A guide to implementation for organisations. National Institute for Health and Clinical Excellence, London, 2005.

3 How to change practice: Understand, identify and overcome barriers to change. National Institute for Health and Clinical Excellence, London, 2007.

To the principles set out in Box 1 could also be added the issue of sustainability.

Whilst the How to …" guides provide common sense advice on what best to do currently, based on interpretation of the available evidence the process of their production also highlighted the scope and size of the research agenda.

2.4. Research into clinical effectiveness

Terminology is a problem in the area of both the practice of, and researching into, clinical effectiveness. The HLG use the term "clinical effectiveness" as it built on the terminology used within the Chief Medical Officer's report. However a study of 33 applied research funding agencies across nine countries identified 29 terms used to refer to some aspect of the processes around clinically effective practice[4]. This confusion has been compounded by the recent prominence of "Translational Research" and the description of the first and second translation gaps. Given the balance of scientific endeavour and funding the term "Translational Research" is mainly thought of as the T1 bench to bedside process of transferring basic science knowledge into new drugs and technologies. Attracting about 1% of the research funding devoted to T1 research the T2 Translational Research is the process of taking current scientific knowledge and ensuring it is applied in routine clinical care.[5]

Within the UK the terms "Implementation" and "Implementation Research" seem to be the best recognised. Therefore, as a focus for its deliberations the CERAG adopted the following definition:

> "Implementation research is the scientific study of methods to promote the systematic uptake of clinical research findings and other evidence-based practices into routine practice, and hence to improve the quality (effectiveness, reliability, safety, appropriateness, equity, efficiency) of health care. It includes the study of influences on healthcare professional and organisational behaviour."
>
> (adapted from Implementation Science http://www.implementationscience.com/info/about/ accessed 10/03/08).

---

4 Tetroe JM, Graham ID, Foy R, Robinson N, Eccles MP, Ward J, Wensing M, Durieux P, Légaré F, Palmhoj Nielson C, Adily A, Porter C, Shea B, Grimshaw J. Health Research Funding Agencies' Support and Promotion of Knowledge Translation: an International Study. Millbank Quarterly 2008: 86;125-155.

5 Woolf SH. The Meaning of Translational Research and Why It Matters. JAMA, 2008; 299: 211 213.

3. The importance of Implementation Research and its funding

Chapter Summary

The findings from clinical and health services research can not change population health outcomes unless health care systems, organizations and professionals adopt them in practice. A consistent finding is that the transfer of research findings into practice is unpredictable and can be a slow and haphazard process.

The relative inattention to implementing what we know is costing lives. There is an imbalance between investment in the development of new drugs and technologies versus improving the fidelity with which care is delivered.

The Cooksey Report suggested an annual research spend (Public sector and major charity) of just over £2 billion. The proportion spent on Health Services (as opposed to biomedical or clinical) Research in general is small. The proportion spent on Implementation Research is impossible to quantify; it is likely to be of the order of a maximum of a few millions of pounds per year.

There have been a number of previous funding programmes for Implementation Research; none are current. The Cooksey Report, having identified the need for implementation and implementation research, offers a sound basis on which to elaborate the implementation research agenda as a core part of a research agenda of key relevance to the NHS.

Internationally, Implementation Research is a recognised area of funding within other healthcare systems.

One of the major problems with not having a clearly identified, named Implementation Research funding stream is that the whole area loses "profile"; the issues become blurred and the central focus of the routine uptake of findings from clinical research programmes into routine care becomes lost to research enquiry.

3.1. Why is Implementation Research important?

3.1.1. There are important gaps in applying clinical evidence in routine healthcare

The findings from clinical and health services research can not change population health outcomes unless health care systems, organizations and professionals adopt them in practice[6].

A consistent finding is that the transfer of research findings into practice is unpredictable and can be a slow and haphazard process. This is demonstrable both within the UK NHS:

6 Grimshaw J, Ward J, Eccles M. Getting research into practice. In: Penchon D, Guest C, Melzer D, Muir, Gray JA, editors. Oxford handbook of public health practice.Oxford: Oxford University Press; 2001.

- The Chief Medical Officer's 2005 report *On the State of the Public Health* contained the chapter "Waste not, want not" which included examples of the mismatch between practice and evidence within the UK National Health Service.
- A review of quality of care studies (including diabetes care) from UK primary care concluded that "in almost all studies the process of care did not reach the standards set out in national guidelines or set by the researchers themselves."[7]
- NICE is producing a lot of guidance. An evaluation of the impact of NICE guidance across nine areas concluded that impact was mixed with effects on the use of drugs or technologies that could be confidently ascribed to the release of the guidance occurring in only two cases.[8]
- The Health Foundation report on quality gaps in stroke care[9] reported figures from the RCP Sentinel Stoke Audit[10] showing rates of achievement against a set of key indicators varying from 54% to 76% with one (on antithrombotic therapy at discharge) achieving 100%.

and more widely in other international healthcare systems:

- Researchers in the US and the Netherlands have estimated that 35-45% of patients are not receiving care according to the scientific evidence and that 20-25% of care provided is not needed or could cause harm.[11][12][13]
- Australia's National Institute of Clinical Studies monitors performance against standards for 11 conditions that span a range of clinical conditions and primary and secondary care.[14] They show varying sized gaps between the evidence and current performance. As they monitor these indicators over time they also chart the changes in the gaps and service and policy initiatives to close them. They demonstrate persisting gaps.

---

7 Seddon ME, Marshall MN, Campbell SM, Roland MO. Systematic review of studies of quality of clinical care in general practice in the UK, Australia and New Zealand. QHC 2001;10(3):152-8.

8 Sheldon TA et al. What's the evidence that NICE guidance has been implemented? Results from a national evaluation using time series analysis, audit of patients' notes, and interviews. BMJ. 2004; 329(7473): 999.

9 Leatherman S, Sutherland K, Airoldi M. Bridging the quality gap: Stroke. The Health Foundation, London, 2008.

10 Royal College of Physicians (2005). National Sentinel Stroke Audit Report, 2004. RCP.

11 McGlynn EA, Asch SM, Adams J, Keesey J, Hicks A, DeCristofaro A, Kerr EA. The quality of health care delivered to adults in the United States. New England Journal of Medicine 2003 ;348:2635-45.

12 Schuster ME, McGlynn E, Brook RH. How good is the quality of healthcare in the Unitd States? Millbank Quarterly 1998;76:517-63.

13 Grol R. Successes and failures in the implementation of evidence-based guidelines for clinical practice. Medical Care 2001;39: 46-54.

14 National Institute of Clinical Studies, Evidence-Practice Gaps Report Volume 1: A review of developments: 2004–2007. Canberra: National Health and Medical Research Council; 2008.

3.1.2. Healthcare professionals and organisations are unlikely to innovate systematically and reliably

In a structured review of healthcare professionals views on clinician engagement in quality improvement Davies et al identified 86 empirical reports relevant to the review.[15] They report that the literature suggests: healthcare professionals are heterogeneous in relation to their definition of quality; their perception of the need for quality improvement; their attitudes to quality improvement initiatives; their attitudes to clinical guidelines and evidence-based practice. In addition, they have a limited understanding of the concepts and methods of quality improvement and quality improvement is often the scene of turf battles. Under the heading of perceived barriers they also stated that "Many of the identified barriers arise from the well-documented problems of working effectively between and across health professions. This means that although more time and more resources may be necessary or helpful (directly and in their explicit recognition of healthcare professionals' concerns), they are unlikely to be sufficient on their own to overcome the substantial barriers to clinicians' active engagement in successful quality improvement".

Healthcare professionals are an important part of the organisation in which they work (and are subject to organisational policies, procedures and cultures) and this review offers a partial explanation for the persistent quality gaps identified in 4.1.1 and also supports the contention that it is unlikely that this will change spontaneously.

3.1.3. The health gains from successful implementation can exceed those of enhancing current technologies

Woolf has argued that the relative inattention to implementing what we know is costing lives. He suggests that there is an imbalance between investment in the development of new drugs and technologies versus improving the fidelity with which care is delivered; he estimates the latter to attract 1% of the resources of the former.[16] In two examples he shows that studies to produce new drugs with enhanced efficacy would fail to achieve the health gains that could be achieved by delivering older agents to all eligible patients.

Examples from Woolf & Johnson (verbatim quote)

Suppose a disease claims 100,000 lives each year and a drug is available that reduces the mortality rate from that disease by 20% (relative risk reduction [RRR] = 0.20). The drug therefore has the potential to save 20,000 lives each year, but if only 80% of eligible patients receive the drug, only 16,000 deaths will be averted. If society made no effort to improve the efficacy of the drug but managed to deliver it to 100% of eligible persons, 20,000 (4,000 additional) lives would be saved. But if society retains the 20% gap in delivery and works to enhance the efficacy of the drug, the RRR

15 Davies H, Powell A, Rushmer R. Healthcare professionals' views on clinician engagement in quality improvement: A literature review. The Health Foundation, London, 2007.

16 Woolf SH, Johnson RE. The Break-Even Point: When Medical Advances Are Less Important Than Improving the Fidelity With Which They Are Delivered. Ann Fam Med 2005;3:545-552.

would have to rise above 25% (100,000 x 0.25 x 0.8 = 20,000) to do as much good (ie, to save 4,000 additional lives).
Delivering the drug to only 60% of patients would save only 12,000 lives (B1), and improving delivery to 100% would save 8,000 additional lives. To save 8,000 additional lives without improving upon the delivery rate of 60% (B2), the RRR of the drug must be increased to at least 33.3%. Developing a more efficacious drug is more beneficial than improving access only if the new relative risk reduction (RRR) exceeds the existing RRR divided by the proportion of the population exposed to treatment.

Antiplatelet Therapy to Prevent Recurrent Stroke

A systematic review by the Antithrombotic Trialists Collaboration reported that the use of aspirin by patients who had previously experienced a stroke or transient ischemic attack reduces the incidence of recurrent nonfatal strokes by 23%. That is, in a population in which 100,000 people were destined to have strokes, 23,000 events could be prevented if all eligible patients took aspirin. McGlynn et al reported, however, that antiplatelet therapy is given to only 58% of eligible patients. At that rate, only 13,340 strokes would be prevented in the hypothetical population, whereas achieving 100% fidelity in offering aspirin would prevent 23,000 strokes (i.e., 9,660 additional strokes). Not addressing the fidelity of aspirin delivery and opting instead to develop better drugs makes sense … only if the newer agents can lower stroke incidence by at least 40% (100,000 x 0.40 x 0.58 = 23,000), but this increased efficacy would require a proportional improvement over aspirin of 74%. The pharmaceutical industry has invested heavily in alternative antiplatelet therapies; clopidogrel and ticlopidine underwent extensive testing in trials involving 22,976 subjects. Rather than demonstrating a 74% improvement over aspirin, however, these drugs were only 10% to 12% more effective in preventing vascular events. It is worth asking whether the resources expended for the antiplatelet trials might have prevented more vascular events if they were invested in better systems for the delivery of aspirin.

Statin Use by Patients With Coronary Artery Disease

The use of simvastatin or pravastatin by patients with coronary artery disease reduces 5-year coronary artery disease mortality by as much as 24%. McGlynn et al report, however, that statins are prescribed to only 33% of eligible patients. Using the logic outlined above, we can posit that developing statins that surpass simvastatin or pravastatin is better for population health than achieving 100% uptake only if the new agents are 3 times as potent, reducing 5-year coronary artery disease mortality by at least 72% (100,000 x 0.72 x 0.33 = 24,000). The degree to which the new generation of statins (eg, atorvastatin, rosuvastatin) lower mortality is unknown, pending the results of ongoing trials, but the evidence regarding their effects on lipid levels suggests that they are not 3 times as potent. Rosuvastatin is only 26% more effective than pravastatin and 12% to 18% more effective than simvastatin in lowering levels of low-density lipoprotein cholesterol. Although this superiority is clinically significant, we conclude

that developing these agents has done less to save lives than would robust delivery systems that bring the older statins to all eligible patients.

A redistribution of research funding away from enhancement of technologies and towards ensuring more appropriate use of existing effective technologies would, he argues, be a more effective use of resources.

3.1.4. Getting clinical evidence into practice; Implementation research – a sizeable evidence base but still a young science

Recognition of quality gaps such as these has led to increased interest in more active implementation strategies. Over the past 10 years a body of implementation research has developed.[17][18][19] This demonstrates that interventions can be effective, but provides less information to guide the choice or optimise the components of such complex interventions in practice.[20] While the effectiveness of interventions varies across different clinical problems, contexts and organizations, studies provided scant theoretical or conceptual rationale for their choice of intervention[21] and only limited descriptions of the interventions and contextual data.[22] Research on economic and political approaches to change is scarce[23] and it is therefore not surprising that little is known about how best to integrate disease and case management interventions into existing healthcare at the system level.

Thus the science of implementation research is still a work in progress, largely due to the fact that it is a relatively young science.

---

17 Bero L, Grilli R, Grimshaw JM, Harvey E, Oxman AD, Thomson MA. Closing the gap between research and practice: an overview of systematic reviews of interventions to promote implementation of research findings by health care professionals. BMJ 1998;317:465-8.

18 Grimshaw J, Thomas RE, Maclennan G, Fraser C, Ramsay C, Vale L, et al. Effectiveness and efficiency of guideline dissemination and implementation strategies. Health Technol Assess 2004;8(6).

19 Grimshaw JM, Shirran L, Thomas RE, Mowatt G, Fraser C, Bero L, et al. Changing provider behaviour: an overview of systematic reviews of interventions. Med Care 2001;39(Suppl 2):II-2-II-45.

20 Foy R, Eccles M, Jamtvedt G, Grimshaw J, Baker R. What do we know about how to do audit and feedback? BMC Health Services Research 2005;5:50.

21 Davies P, Walker A, Grimshaw J. Theories of behaviour change in studies of guideline implementation. Proceedings of the British Psychological Society 2003;11(1):120.

22 Grimshaw JM, Thomas RE, MacLennan G, Fraser C, Ramsay CR, Vale L, et al. Effectiveness and efficiency of guidline dissemination and implementation strategies. Health Technol Assess 2004;8(6):1-84.

23 Grol R, Grimshaw J. From best evidence to best practice: effective implementation of change in patients' care. Lancet 2003;362:1225-30.

3.2. Patterns of research funding in the UK

Whilst precise, up to date, figures are difficult to obtain, the following figures, taken from the Cooksey Report[24] are illustrative of the profile of research funding. Although precise amounts may have varied, the overall proportions are unlikely to have changed.

**Public sector research funding**
NHS R&D (2006–07) £753 million (including £50 million of capital and the DH Policy Research Programme)
MRC (gross expenditure for 2005–06) £525.8 million
Scotland's Chief Scientist Office (2006–07) £60 million (includes £43 million for research infrastructure costs)
Northern Ireland HPSS R&D Fund, £12 million per annum.
Wales Office of Research & Development, £22.8 million
**Research charities (the two largest individual funders)**
The Wellcome Trust, £400 million
Cancer Research UK £250 million

Together these figures amount to an annual research spend of just over £2 billion. These figures do not take any account research spend by the private sector, particularly the pharmaceutical and the devices industries.

The proportion spent on Health Services (as opposed to biomedical or clinical) Research in general is small; it varies by funder with <2% of MRC expenditure to about 15% of NHS R&D spend.

The proportion spent on Implementation Research is impossible to quantify; it is likely to be of the order of a maximum of a few millions of pounds per year – even assuming it was at the level of £2.5 million a year this would represent 0.125% of the public sector and major charity annual research spend.

With this profile of research spending it is reasonable to argue that the UK is in exactly the same position as Wolff (ref 16 above) describes for the US and it is likely that UK citizens are suffering avoidable mortality and premature mortality as a result of the imbalance of research funding.

3.3. Previous funding for Implementation Research

3.3.1. The NHS R&D Implementation Methods Programme

The most co-ordinated UK based previous attempt at funding Implementation research was the Implementation Methods Programme. The following description is drawn, often

24 Cooksey D. A review of UK health research funding. HMSO, Norwich, 2006.

verbatim, from the evaluation that was commissioned from the Health Economics Research Group at Brunel University.[25][26]

Concern with research implementation was a major factor behind the creation of the NHS R&D Programme in 1991. In 1994 an Advisory Group was established to identify research priorities in this field. The Implementation Methods Programme (IMP) flowed from this and its Commissioning Group funded 36 projects. Funding for the IMP was capped before the second round of commissioning. The Commissioning Group was disbanded and eventually responsibility for the programme passed to the National Co-ordinating Centre for NHS Service Delivery and Organisation R&D (NCCSDO).

The IMP had been established with a budget of about £8 million. Half was allocated in the first round, although £534K of that was not spent after one study was terminated following the feasibility stage. Prior to the second round a funding cap on national R&D programmes was announced and in that round just £800K was allocated to three projects.

The more traditional two-stage process resulted in 149 outline applications. Eventually 69 full applications were peer reviewed and assessed by the Commissioning Group, but their success rate 'was low and particularly disappointing' (NHS Executive, 1996) and Commissioning Group members were reported to be disappointed that the ideas from the outline stage were often not sufficiently developed in the full applications. There were concerns about many of the quantitative and qualitative proposals; these were often methodological and included issues such as the overall research approach, the underlying theoretical basis and the analytical strategy.

The reports conclusions state: "We conclude that the IMP was seen by many of those involved as a new and exciting field. Looking back, they were generally positive about what was started through the IMP. It commissioned a series of projects that produced some important, rigorous, and cutting edge research, at least some of which is making an impact. But this is a complex area in which traditional clinical research, health services research and the social sciences all have a role to play. A unique set of difficulties, as well as opportunities, was faced by those responsible for taking the programme forward. The intellectual challenges of constructing a programme to cover such a vast area with diverse and sometimes conflicting conceptual and methodological perspectives, were compounded by practical problems. These included the capping of the programme's funding and the premature winding up of the Commissioning Group. As a result, this complex programme, which arguably needed better support than its more clinically orientated predecessors, did not receive it at some stages. Those involved in the programme had a considerable task – the difficulties of which were not completely appreciated at the time. They are clearer in retrospect and feed into the lessons and recommendations presented here, but it is recognised that a programme such as the SDO is already adopting some of the steps.

---

25 Hanney S, Soper B, Buxton M. Evaluation of the NHS R & D Implementation Methods Programme. HERG Research Report No. 29, August 2003. Health Economics Research Group, Brunel University, Uxbridge, Middlesex UB8 3PH, UK. http://www.brunel.ac.uk/depts/herg/pubs/internal.html.

26 Soper B, Hanney SR. Lessons from the evaluation of the UK's NHS R&D Implementation Methods Programme. Implementation Science 2007, 2:7 doi:10.1186/1748-5908-2-7.

Other recommendations are more specifically relevant when the SDO Programme is considering an area such as implementation methods research. It would be desirable for more time to be made available for preparatory work than was allowed for the IMP and also scope provided for the programme to be able to re-visit issues and learn from early results. It is difficult to incorporate all the analysis that is required if a programme is operating in a time-limited way.

The reports conclusion that research implementation is a crucial area for the NHS R&D Programme leads to the recommendation that more R&D activity is needed in this field in order to assist delivery of some key NHS agenda items."

One of the key achievements of the Implementation Methods Programme was the way that it increased the profile of implementation both within the research community and the NHS.

3.3.2. The MRC Implementation research call

In 2006 MRC issued a joint call for implementation and methodology applications via the Health Services and Public Health Research Board. The overall budget for the call was £10 million with £1 million earmarked for Implementation Research. Projects were given an indicative maximum budget that they could bid for. The MRC Health Services and Public Health Research Board has subsequently been disbanded.

3.3.3. Other funding for Implementation research

The SDO Programme is sometimes thought of as the home of implementation research. However, within the SDO Programme the focus to date has largely been on generating new knowledge about how best to organise service delivery – an entirely appropriate aim, and the SDO equivalent of generating knowledge about clinical interventions from clinical trials. Alongside this work, the SDO Programme has recently identified as a key priority area the commissioning of new research on knowledge mobilisation. While, in keeping with the remit of the SDO Programme, this new work will focus especially on how knowledge gets generated and used in managerial practice; there will inevitably be issues that arise about, for example, how mangers can set an organisational context within which clinical effectiveness and quality improvement activities can flourish.

Running alongside the SDO programme of research commissioning is a new work stream on knowledge mobilisation and capacity building for the service, to ensure that SDO outputs and other related knowledge are used to best effect. Parallel evaluation of these activities is also likely to feed into the implementation research agenda.

A further relevant strand of National Institute for Health Research (NIHR) activities relates to the recently funded Collaborations for Leadership in Applied Health Research and Care (CLAHRC). Each of these Collaborations has integral to it significant work on implementation of emergent clinical research findings with parallel evaluation activities.

The Health Foundation (from its website) has what should be an interest in Implementation research – "In order to improve the quality of healthcare, we need to build our understanding of what works to improve quality and performance and what professionals and patients think about the healthcare they respectively deliver and receive." It has invested in QQUIP – Quest for Quality and Improved Performance –

which is a five-year, £2.5 million research initiative to provide an evolving quality of care resource for health services. Between 2003 and 2005, The Health Foundation conducted a series of surveys of patients' opinions and attitudes towards the healthcare they receive in the UK and beyond. Finally they have recently commissioned RAND Europe to assess the UK-wide picture of clinical engagement and quality improvement in healthcare.

It is likely that other research funders fund pieces of work that would be considered Implementation Research but this is not an explicit area of research interest for any of them.

3.3.4. The Cooksey Report and Implementation Research

The Cooksey Report was a major influence in re-shaping the structures of research funding within the UK. In terms of Implementation Research it laid the foundation for further elaborating the importance of implementation research. It talked of the implementation of research findings as the "second gap in translation" (the relevant section of report is in Appendix 2). The report follows the North American use of the term Translational Research to apply to both "bench to bedside" and the uptake of clinical research findings into routine practice, a problem discussed by Woolf[27]. The Cooksey Report devotes most of its attention to the first gap in translation; for the second gap in translation it helpfully identifies that the HTA programme

> "should benefit significantly from a greater proportion of support for clinical research, ***quality and safety research*** and public health intervention research within the overall portfolio of UK health research. There is a crucial need for improved Knowledge Transfer, Reception and Use of HTA findings through the NHS" (our bold italics).

Whilst the report also highlights the success of the SDO Programme and suggests that

> "plans to enlarge the programme should be developed in parallel with those for further expansion in the capacity of the NHS for Knowledge Transfer, Reception and Use."

It completes the paragraph with

> "The National Programme for Information Technology, operated by NHS Connecting for Health, may be an important first step in the evolution of ***decision-based software systems*** that healthcare professionals will need in order to manage effectively the growing scale of our knowledge base and thus improve the care of patients. However, this programme is not likely to be operational at such a level for some time. Nor has it been designed specifically for the purpose of Knowledge Transfer. In any event, the uptake of new knowledge, particularly complex information, often depends on the ***direct communication of new potential users with those familiar with that new technology***, so that solutions

27 Woolf SH. The Meaning of Translational Research and Why It Matters. JAMA, 2008; 299: 211 213.

> involving Information Technology are unlikely to be entirely sufficient. Likewise, ***direct marketing*** or ***information campaigns*** are unlikely to adequately support the spread of these types of knowledge within the health services. (our bold italics)

By talking about implementation interventions (bold italics) in this way the Report sets the stage for this report to further highlight the fact that there are relevant systematic reviews on the effectiveness of these and that their further study (in terms of effectiveness, configuration, delivery etc.) should be a core interest of the NIHR.

It also suggests the need for a greater responsiveness to the research recommendations from NICE – a major body concerned with implementation (in England and Wales).

However, whilst having expressed the principle of more quality and safety (i.e. implementation) research, it suggests

> "a pilot programme … to examine the effectiveness of employing a small number of full-time ***'Knowledge Transfer Champions'*** to disseminate the findings of health services research and facilitate early adoption of those findings into routine practice in the NHS"

As a variation of the current fashion for Knowledge Brokers, this would represent an untried intervention but one that could be easily evaluated within an Implementation Research funding programme.

Thus, the Cooksey Report, having identified the need for implementation and implementation research, offers a sound basis on which to elaborate the implementation research agenda as a core part of a research agenda of key relevance to the NHS.

3.4. Implementation research funding internationally

Internationally, Implementation Research is a recognised area of funding within other healthcare systems – for example the USA (AHRQ), Canada (KT Programme within CIHR) and Australia (National Institute of Clinical Studies within the National Health and Medical research Council).
Recognising that research funders have roles within their activities in relation to implementation Tetroe et al.[28] conducted a study "to determine the knowledge translation policy, expectations, and activities of health research funding agencies both in Canada and internationally" asking thirty-three agencies from Australia, Canada, France, the Netherlands, Scandinavia, the United Kingdom, and the United States about their role in promoting the results of the research they fund.
They suggested that:

> "Research funders could promote knowledge translation in a number of ways, as they are in a position to influence researchers' knowledge translation activities. Funders could emphasize the importance of knowledge translation as an integral

---

[28] Tetroe JM, Graham ID, Foy R, Robinson N, Eccles MP, Ward J, Wensing M, Durieux P, Légaré F, Palmhoj Nielson C, Adily A, Porter C, Shea B, Grimshaw J. Health Research Funding Agencies' Support and Promotion of Knowledge Translation: an International Study. *Millbank Quarterly* 2008: 86;125-155.

> part of the research process and require that it be addressed in the grant proposal, and they might require researchers to work with end users (e.g., policymakers, clinicians) as partners in writing the grant proposal and in conducting and eventually implementing the research. Research funders might also promote KT directly by developing their own knowledge translation strategy, disseminating information about funded and completed research, involving end users in prioritizing research topics (i.e., commissioned research), ***and funding implementation research (i.e., the scientific study of methods to promote the use of research findings in practice)***." (our emphasis)

Using semi-structured interviews with key informants supplemented by information from organisations websites they found

> "There was a lack of clarity between agencies as to what is meant by KT and how it is operationalised. Agencies also varied in their degree of engagement in this process. The agencies' abilities to create a pull for research findings; to engage in linkage and exchange between agencies, researchers, and decision makers; and to push results to various audiences differed as well. Finally, the evaluation of the effectiveness of KT strategies remains a methodological challenge."

So, not only is the problem of (failure of routine) implementation an international phenomenon, so are the problems in funding implementation research, a sentiment echoed by the Cooksey Report

> "The Review has found that the impact of health research in the international context is constrained by a lack of coordination between funders. The Review supports the recommendation by the Government's Chief Scientific Adviser that a forum should be set up to facilitate collaboration on development research in the UK."

3.5. The profile of Implementation Research within NIHR

One of the major problems with not having a clearly identified, named Implementation Research funding stream is that the whole area loses "profile"; the issues become blurred and the central focus of the routine uptake of findings from clinical research programmes into routine care becomes lost to research enquiry.

The notion of researching the implementing of new knowledge from clinical or service delivery research as a coherent area of scientific enquiry has been largely lost to the research agenda. Considering the potential health gains from implementation research that Woolf identified, this loss is costing UK citizen lives.

In countries where there is a named, dedicated, funding stream (e.g. Canada, Australia) the research area has a higher profile with both researchers and with clinicians. There is the potential for senior researchers to establish programmes of research (rather than doing one off studies), junior researchers to make it a career choice, and clinicians become willing collaborators thereby facilitating the spread of knowledge and the improvement of methods.

4. Specific considerations for an Implementation Research Agenda

Chapter Summary

In elaborating the Implementation Research agenda we identified a number of important overarching considerations that should influence thinking about and commissioning Implementation Research.

It is important to consider the *multiple levels* at which healthcare is delivered and the *interplay* between them in their cultural context.

Implementation research centrally involves the study of changing behaviour and maintaining change – in organizations and the groups and individual healthcare professionals within them.

The use of theory in Implementation Research offers (at least) three important potential advantages. Theories offer a generalisable framework that can apply across differing settings and individuals; they offer the opportunity for the incremental accumulation of knowledge; and they offer an explicit framework for analysis.

Frameworks are potentially useful tools for considering the issues that a research agenda needs to address. Inevitably there is no one ideal, universally accepted framework that will fit all purposes; different frameworks will often reflect different purposes, disciplinary or philosophical standpoints and so will appeal to different groups or individuals.

A general complaint of implementation studies (often trials) is that the need for experimental control, maximising internal validity, compromises external validity. As ever, the balance of considering these two dimensions of validity depends on the question that is being answered at the time.

4.1. Levels of engagement - seeing the whole picture

Efforts to improve the quality of care, particularly for chronic diseases which are complex to manage, need to occur at and be coordinated across multiple levels – such as the patient, clinician, team, organisation, system, and population. In their article in Milbank Quarterly[29] Ferlie and Shortell said:

"Fuelled by public incidents and growing evidence of deficiencies in care, concern over the quality and outcomes of care has increased in both the United Kingdom and the United States. Both countries have launched a number of initiatives to deal with these issues. These initiatives are unlikely to achieve their objectives without explicit consideration of the ***multilevel approach to change that includes the individual, group/team, organization, and larger environment/system level****. Attention must be given*

[29] Ferlie EB, Shortell SM. Improving the quality of health care in the United Kingdom and the United States: a framework for change. The Milbank Quarterly 2001;79(2):281-315.

to issues of leadership, culture, team development, and information technology at all levels. A number of contingent factors influence these efforts in both countries, which must each balance a number of ***tradeoffs between centralization and decentralization*** *in efforts to sustain the impetus for quality improvement over time. The multilevel change framework and associated properties provide a framework for assessing progress along the journey."* (our bolding).

This highlights the importance of considering the *multiple levels* at which healthcare is delivered and the *interplay* between them in their cultural context.

4.2. Behaviour change

At the risk of stating the obvious, implementation research centrally involves the study of changing behaviour and maintaining change – in organizations and the groups and individual healthcare professionals within them. It concerns the study of behaviour, the determinants of behaviour and how to change and maintain behaviour with due cognisance of the organisational context within which behaviour is enacted.

This is worth emphasizing because there is often confusion between the study of behaviour and its determinants and the study of changing behaviour. This distinction is important and it has a series of knock-on consequences for the design and conduct of studies in terms describing (often complex) interventions, describing the important elements of care delivered in the control group, clarifying the "active ingredients" of interventions, defining outcomes, considering measurement of process variables and calculating effect sizes. There is a science of behaviour change and this should be drawn on when designing, conducting and evaluating implementation research. One obvious corollary of this is that implementation research must draw on a wide variety of research disciplines including (but not limited to): psychology, sociology, anthropology, economics, organisational studies, information science and others.

4.3. Theories, Frameworks and Models

4.3.1. Theories

The use of theory in Implementation Research offers (at least) three important potential advantages. Theories offer a generalisable framework that can apply across differing settings and individuals; they offer the opportunity for the incremental accumulation of knowledge; and they offer an explicit framework for analysis. The evaluation of the Implementation Methods R&D Programme identified the Commissioning Group's observation of the lack of underpinning theory across a range of the applications submitted.

There is no clear agreement about what makes a study or an intervention "theory-based" means leading to a range of phrases such as "informed by theory", "underpinned by theory", "theory-inspired" and "theory-based". In the absence of anything better, within this report we have stayed with "theory-based", however imprecise this may be. There is also little agreement about which theories are key and under what circumstances; associated with this there is considerable overlap between theories. In the process of developing a theoretical framework for implementation research a group of health

psychologists and health services researchers used a consensus process reduced 33 theories containing 128 constructs to a core set of 12 construct domains.[30]

There is a surprising lack of explicitly theory-based intervention studies within the field of Implementation Research. Within the most recent review of guideline implementation[31], the authors of included studies provided an explicit theoretical rationale for their intervention in less than 10% of studies.[32] Theory-based studies predicting or evaluating change at an organisational level are also uncommon. Bower et al demonstrated in a UK study that a mixture of theoretical - team climate - and structural factors explained 31% of the variance in diabetes management.[33] Estabrooks et al explored the influence of individual and organisational (context determined by the PARIHS framework[34]) level factors on research utilization in a (large) sample of Albertan nurses - using modelling - found that mainly individual factors contributed to likelihood of using research, with some of the variance explained by organisational level factors.[35]

Given this absence both of a theoretical underpinning and of interventions attempting to explicitly and prospectively modify theoretical constructs, it can be difficult to interpret across implementation studies why interventions have had positive or negative effects.[36]

The CERAG agreed that appropriate consideration of theory was an important element of Implementation Research. As well as a more thoughtful use of theory there is a need to work through the various stages of using theory and resolving such apparently simple issues as what it means for an intervention to be theory based or what is the theoretical basis of behaviour change.

---

30 Michie S, Johnston M, Abraham C, Lawton R, Parker D, Walker A on behalf of the ''Psychological Theory'' Group. Making psychological theory useful for implementing evidence based practice: a consensus approach. Qual Saf Health Care 2005;14:26–33.

31 Grimshaw JM, Thomas RE, MacLennan G, Fraser C, Ramsay CR, Vale L, Whitty P, Eccles M, Matowe L, Shirren L,Wensing M, Dijkstra R, Donaldson C. Effectiveness and efficiency of guideline dissemination and implementation strategies. Health Technol Assess 2004;8:1–72.

32 Davies P, Walker A, Grimshaw J. Theories of behavior change in studies of guideline implementation. Proc Br Psychol Soc 2003;11:120.

33 Bower P, Campbell S, Bojke C, Sibbald B. Team structure, team climate and the quality of care in primary care: an observational study. Quality and Safety in Health Care 2003; 12(4):273-279.

34 Rycroft-Malone, J. Kitson, A. Harvey, G. McCormack, B. Seers, K. Titchen, A. & Estabrooks, C. (2002) Ingredients for change: Revisiting a conceptual framework, Quality and Safety in Health Care, 11, 174 – 180.

35 Estabrooks CA, Midodzi WK, Cummings GG, Wallin L. (2007) Predicting research use in nursing organizations: a multi-level analysis. Nurse Res, 56(4 Suppl): S7-23.

36 Foy R, Eccles M, Jamtvedt G, Young J, Grimshaw J, Baker R. What do we know about how to do audit and feedback? Pitfalls in applying evidence from a systematic review. BMC Health Services Research 2005, 5:50. http://www.biomedcentral.com/1472-6963/5/50

4.3.2. *Frameworks and Models*

Frameworks are potentially useful tools for considering the issues that a research agenda needs to address. Inevitably there is no one ideal, universally accepted framework that will fit all purposes; different frameworks will often reflect different purposes, disciplinary or philosophical standpoints and so will appeal to different groups or individuals. A recent review identified 31 “planned action theories”.[37]

However, all these change models have a number of key features in common. In relation to the introduction of clinically effective practice they all require:

1. an understanding of current practice
2. an understanding of the reasons for mis-matches between current behaviour and the evidence
3. an understanding of attributes of individuals responsible for introducing the clinically effective practice and the context(s) in which the individuals operate (including patient, team and organizational factors)
4. the current evidence of the effectiveness of strategies to introduce changes in behaviour
5. the most appropriate match between facilitators (to be strengthened)/barriers (to be weakened) and behaviour change strategies
6. the introduction and evaluation of the effectiveness of the behaviour change strategy

Three archetypal research use models (summarised below) were inductively derived from studies in social care and described by Nutley et al.[38]

- The *research-based practitioner model*, where research use is the responsibility of individual practitioners;
- The *embedded research model*, where research use is achieved by embedding research in the systems and processes of service delivery; thus it is the service managers and policy-makers who play a key role;
- The *organisational excellence model*, where the key to successful research use lies in the development of appropriate structures, processes and cultures within local service delivery organisations.

---

37 Graham ID, Tetroe J, and the KT Theories Research Group. Some Theoretical Underpinnings of Knowledge Translation. Academic Emergency Medicine 2007; 14:936–941.

38 Nutley SM, Walter I, Davies HTO. *USING EVIDENCE: How research can inform public services*. The Policy Press, Bristol 2007. Chapter 7

Research-based practitioner model. In this model:

- It is largely the role and responsibility of the individual practitioner to keep abreast of research and ensure that it is used to inform day-to-day practice;
- The use of research is most often seen as a linear process of accessing, appraising and applying research in largely instrumental ways;
- Practitioners usually have high levels of professional autonomy to change practice based on their interpretation of research findings;
- Professional education and training are seen as the key factors in enabling research use;
- Access provisions to knowledge resources are a common preoccupation.

Embedded research model. In this model:

- Research use is largely achieved by embedding research in the systems and processes of practice, by way of standards, policies, procedures and tools;
- Responsibility for ensuring research use lies primarily with policy makers (national, regional and local) and local service delivery managers;
- The use of research is largely seen as both a linear and an instrumental process: research is translated directly into planned practice change;
- Funding, performance management and regulatory regimes are used to encourage or coerce the use of research-based guidance and tools.

Organisational excellence model. In this model:

- The key to successful research use largely rests with local service delivery organisations: their leadership, management and organisational arrangements;
- Research use is supported by developing an organisational culture that is "research-minded";
- There will usually be significant local adaptation of research findings and ongoing learning within teams and local organisations;
- Partnerships with local universities and intermediary organisations may be used to facilitate both the creation and use of research knowledge;
- The nature of the research considered may be much wider than instrumental 'what works' findings.

4.4. Balancing internal and external validity

A general complaint of studies (often trials) is that the need for experimental control, maximising internal validity, compromises external validity. As ever, the balance of considering these two dimensions of validity depends on the question that is being answered at the time. However, in an area of research where the impact of context is often core to understanding it is important for a research agenda to appropriately recognise and address both internal and external validity though the balance between these may vary. Early in the process of developing and evaluating an intervention it may be most appropriate to evaluate whether an intervention can work in relatively highly

controlled settings. It is often the case that such studies can be smaller than subsequent evaluations. Assuming this to be the case it is then appropriate to move on to evaluating the impact of the intervention in a real world (i.e. less tightly prescribed) setting.

The dichotomisation of studies in this way, into efficacy and effectiveness studies is useful in informing design in terms of the focus on internal or external reliability. In reality it is a spectrum – sometimes referred to as the explanatory/pragmatic spectrum. In explanatory studies the focus is on efficacy with validated interventions and high internal validity often in a selected population. In pragmatic trials the focus is on external validity (generalisability) of a strategy in the full clinical population of interest usually with an economic evaluation along side. Whichever is the focus, both are legitimate subjects of enquiry within a research agenda and both will be of interest to users of research.

A thorough treatment of internal and external validity is given in Shadish et al's book[39].

---

39 Shadish, Cook, Campbell. Experimental and quasi-experimental designs for general causal inference. Houghton Mifflin, Boston 2002.

5. The research agenda

Chapter summary

This discussion of the research agenda is aimed primarily at commissioners of research but will also be of relevance to a broader range of policy makers and researchers.

Many of the research areas are interlinked. Within the chapter we offer exemplar questions within each of them in order to illustrate key issues.

- The research areas are:
  - Context;
  - Behavioural determinants and evaluation of change strategies;
  - Testing of theory in implementation research;
  - Knowledge attributes and knowledge generation
- Cross cutting issues
  - Methodology;
  - Implementation Research across different areas of clinical practice;
  - Knowledge infrastructure for Implementation;
  - Sustainability
- Communication strategy/engagement with the NHS
- Workforce issues
  - Capacity to do implementation;
  - Capacity to do implementation research
- Attributes of research teams addressing this agenda
- Implementation and evidence of benefit from clinical and public health interventions

5.1. Who is this research agenda aimed at?

This discussion of the research agenda is aimed primarily at commissioners of research but will also be of relevance to a broader range of policy makers and researchers.

Whilst this report has been discussed and written in the context of the UK National Health Service and the National Institute for Health Research (NIHR) it is possible that a variety of other research commissioning organisations could use it to identify areas that are a priority for them. However, it has been considered in its entirety and, in terms of

programmatic commissioning, a piecemeal approach to addressing it could leave important areas unaddressed.

5.2. Research areas

Many of these research areas are interlinked. We offer exemplar questions within each of them in order to illustrate key issues. The processes suggested in the subsequent recommendations will further elaborate and prioritise the content of this agenda.

5.2.1. Context

The impact of context on implementation is important and systematic study of the attributes of context (and their role and modifiability) that form barriers or facilitators to implementation is needed.

The responsiveness of context is important to understand (and influence) culture and other attributes of organisations and the individuals within them and their interests related to implementation of new knowledge.

The role of context in intervention development needs to be better understood.

5.2.2. Behavioural determinants and evaluation of change strategies

Successful implementation of new knowledge should be built on an understanding of the determinants of behavioural change and maintenance of behavioural change in individuals and organisations. Such understanding would allow the rational development and testing of implementation interventions.

This should include the systematic development and trialing of interventions across a range of conditions and NHS settings. These could include the study of the organisational embedding of new interventions, the effectiveness of healthcare system interventions as well as evaluation of delivering new models/methods of care.

There is a need for studies examining the methods of optimising the content and methods of delivery of interventions.

Evaluations would use a range of (and often a combination of) research designs and methods (e.g.: cluster randomized trials, quasi experimental designs, and qualitative studies.

5.2.3. Testing of theory in Implementation Research

Theory is underused in implementation research. There needs to be considerable work on understanding available theories, on the testing and development of theories and on how to operationalise theory. This work should not be restricted by disciplinary perspectives, worldview or area of application.

5.2.4. Knowledge attributes and knowledge generation – features related to uptake

Research is needed on the important attributes of new knowledge and how these influence its uptake (or not). This would include the attributes of and applicability of what is regarded as evidence by different individuals and in different contexts.

Decision makers have problems accessing, appraising, adapting and applying research evidence. The increasing recognition that implementation of evidence from individual studies may be misleading due to bias in their conduct or random variations in findings, has led to greater emphasis on knowledge syntheses as the basic unit of implementation. Knowledge syntheses interpret the results of individual studies within the context of global evidence thus increasing the 'signal to noise ratio' of implementation activities and increasing the likelihood of their success. Knowledge syntheses provide the evidence base for other implementation vehicles such as patient decision aids, clinical practice guidelines or policy briefs.

Systematic review activities (guided by relevant theory) need to be systematically supported to ensure their continued development. Important areas activity include: compiling and maintaining a register of systematic reviews of implementation research; updating overviews of reviews of professional behaviour change interventions; conducting systematic reviews of methods to improve the implementation of clinical research findings in routine settings; workshops on conduct and use of knowledge syntheses targeted to different stakeholders.

5.3. Cross cutting issues

5.3.1. Methodology

Across all of the areas above there will be important methodological issues that need to be identified, investigated and resolved. These include:

- The area of implementation research needs a common understanding of terms. Important areas of research include: the development of one or more taxonomies of barriers to implementation, mediating mechanisms and pathways; standardised measurement approaches for key elements of the taxonomy; a suite of reporting guidelines for different types of implementation research.
- All of the areas pose measurement challenges such as the development of process and outcome methods and measures for relevant constructs.
- Is there a "core set" of measures that will be applicable to most settings or is each combination of patient team and organisation conceptually unique? The idea of a core set of measures offers greater potential for accumulation of knowledge.
- What are the pros and cons of using proxies for behaviour such as written or web-based vignettes that simulate clinical behaviours?
- The incorporation of economic analysis within implementation research is not necessarily methodologically challenging but it is very uncommon and should be encouraged and supported.

- An explicit examination of the pros and cons of the use of routinely available data to assess implementation: this would include the availability of data; the specificity of data in relation to the implementation of research evidence. Are there situations where there is sufficient routinely available data for economic modelling to demonstrate the viability or otherwise of certain behaviour change strategies? How complex can/should such modelling become?

5.3.2. Implementation Research across different areas of clinical practice

Implementation research will be conducted in a range of clinical areas. This needs to be done in a way that ensures contribution to our incremental understanding of implementation. Research in one clinical area should generate ideas and understanding that can be drawn on in other clinical areas.

5.3.3. Knowledge infrastructure for Implementation

This links to *Knowledge attributes*, (5.2.4 above) and is addressed in the UK by initiatives such as the NHS National Library for Health, the Cochrane Collaboration and Social Care Online. Nonetheless, the process recommended below could formally set out the knowledge infrastructure for implementation. This would be an would be an important exercise in making explicit the content of an infrastructure (staff, skills, resources), its scale and its degree of current (and future) integration into routine healthcare.

5.3.4. Sustainability

The consideration of sustainability permeates the research agenda. It is important to have NHS workforce that can sustain implementation in the clinical setting as a matter of routine. It is important that we learn more about the organisational/contextual factors that enable the sustained use of evidence in practice. It is also important to have a research workforce that can sustain the area of research.

Within research itself it is important to examine attributes of sustainability (within individuals, teams and organizations) and to develop methods to examine whether the effects of interventions are sustained over time.

5.3.5. Communication strategy/engagement with the NHS

As part of integrating implementation and implementation research within the NHS it will be vital to develop an explicit communication and engagement strategy.

5.4. Workforce issues

5.4.1. Capacity to do implementation

How should the NHS workforce (clinicians/practitioners and managers) be trained (undergraduate and postgraduate) in order to optimise their ability to implement new knowledge (without doing harm, overspending, giving more to one patient than another, whilst also stopping doing ineffective things)?

What are effective engagement strategies to involve the workforce in implementation?

What are the important attributes of the workforce that enhance knowledge use and implementation in healthcare settings?

How can these attributes be sustained both within individuals and organisations?

5.4.2. Capacity to do implementation research

Capacity to do research into implementation is limited both within the UK and internationally. The NIHR needs a strategy of building capacity at all levels of the researcher career. Given the time that it takes to build experience in this area NIHR needs a cadre of experienced senior investigators who can direct programmes of research.

A funding strategy should also build junior researchers capable of developing into independent researchers (this should be linked with experience implementation researchers). This could involve a mix of PhD studentships and Fellowship awards.

5.5. Attributes of research teams addressing this agenda

Addressing this research agenda will be an inherently multi and inter-disciplinary endeavor. No one practice or academic group or discipline will bring all the necessary attributes to address the research agenda. The range of required disciplines will vary within and across the various areas of the research agenda but is likely to include some of implementation research, sociology, health psychology, health economics and statistics.

5.6. Implementation and evidence of benefit from clinical and public health interventions

It will most often be the case that the Implementation Research agenda will be applied to areas where there is a clear understanding of appropriate clinical care or public health practice. In some areas there will be insufficient published evidence to inform a clear, shared understanding of optimum practice; in such instances the research agenda should address the need for evidence of efficient clinical and public health practice.

6. Recommendations

1. NIHR should initiate a process to establish a research programme within NIHR with an explicit dedicated, ring-fenced, funding stream for funding Implementation Research.
    a. This process should detail issues such as:
        i. the scope and prioritization of topics for such a programme;
        ii. the potential overlap with current national research programmes within and outwith NIHR;
        iii. the potential overlap with other NIHR funded initiatives – NELH, CLAHRCS, Cochrane Collaboration
        iv. the relevant stakeholders in the process;
        v. the appropriate configuration of such a programme of research – either as a single entity (maximising focus, scare researcher resources and critical mass) or as a dimension of each of the current national programmes (more diffuse but probably more administratively straight forward to establish)
        vi. the establishment of a commissioning group with appropriate expertise to evaluate proposals;
        vii. the timescale for establishing launching and commissioning research within such a programme;
        viii. relevant indicators of success for such a programme to allow its evaluation.
    b. Given the scale for return on investment and potential to save lives this should aim to achieve a steady state annual spend of 2-3% of NIHR total research spend. With total spend estimated at £750 million this equates to approximately £15 - 22 million.
    c. Spending on this scale will not be achievable immediately and so the process should consider an escalating funding process starting at a lower level and incrementally rising to the steady state figure over a number of years.
    d. Long term commitment is needed to deal with the issue of creating a climate conducive to conducting implementation research and the closely linked area of using research findings in routine settings. Without this being seen as both central and important it is unlikely to be sustained.
    e. Consideration should be given to the idea of establishing one or more Centres of Implementation research Excellence along the lines of the Public Health Centres of Excellence.
2. A mix of Project and Programme funding would allow studies of a shorter and more "worked through" nature as well as series of interlinked conceptual, methodological work that is needed in the area.

3. The process of commissioning should be a mix of commissioner defined and curiosity driven. In such a relatively young area it is unlikely to be possible for a commissioned research process to fully cover all relevant areas, particularly in the areas of methodological and conceptual work.
4. In order to enhance capacity development a proportion of the funding should be directed towards studentships, fellowships and bursaries.
5. There should be consideration of the development of training programmes for implementation researchers. Although not a research budget cost there should also be consideration of the development of (pre and post-registration) training programmes for clinicians and non-clinicians within the NHS around building capacity to better use implementation (and clinical) research in daily practice.
6. Implementation research and implementation researchers need to be embedded within the NHS. One way to achieve this would be to consider further strengthening and extending the implementation research dimensions of the CLAHRCS centers. This should also consider how to closely ally those researching implementation with those doing implementation on a daily basis.
7. In order to promote advancement of the research area funding should be directed towards providing opportunities for scientists and clinicians to meet to discuss relevant issues – akin to the ESRC Seminar Series Grants.
8. NIHR should give consideration to establishing a standing advisory group, with appropriate expertise, to continue to develop, oversee, and advise on implementation research within the NHS. Such a body could also make links with other national centres to form an international network.

Appendix 1. Membership of the Clinical Effectiveness Research Agenda Group

Chair

Martin Eccles (Professor of Clinical Effectiveness and the William Leech Professor of Primary Care Research, Newcastle University)

Members

David Armstrong**,** Professor of Medicine and Sociology, Division of Health and Social Care Research, Kings College London

Richard Baker, Professor of Quality in Health Care, Department of Health Sciences, University of Leicester

Kevin Cleary, Medical Director, National Patient Safety Agency

Huw Davies, Professor of Health Care Policy and Management, School of Management, University of St Andrews

Stephen Davies, Director, National Institute for Health Research Service Delivery and Organisation Programme

Paul Glasziou (Professor & Director, Centre for Evidence-Based Medicine, Department of Primary Health Care, University of Oxford)

Irene Ilott, Research Forum for Allied Health Professions and Research Associate, SDO Protocols Based Care Project, Institute of Work Psychology, University of Sheffield

Ann-Louise Kinmonth, Professor of General Practice, General Practice and Primary Care Research Unit, University of Cambridge

Gillian Leng, Implementation Director, National Institute for Health and Clinical Excellence

Stuart Logan, Professor of Paediatric Epidemiology, Director, Institute of Health Service Research, Peninsula Medical School

Theresa Marteau, Professor of Health Psychology, Kings College, London

Susan Michie, Professor of Health Psychology, Centre for Outcomes Research, University College London

Hugh Rogers, Senior Associate, Service Transformation, NHS Institute for Innovation and Improvement, University of Warwick

Jo Rycroft-Malone, Reader in Health Services Research, College of Health & Behavioural Sciences, University of Wales

Bonnie Sibbald, Professor of Health Services Research, National Primary Care R&D Centre, University of Manchester

Secretariat

Dierdre Feehan, Department of Health.

Appendix 2 Selected sections from the Cooksey Report relating to the second gap in translation.

7.46 An equally crucial stage in translating research into practice is the evaluation and identification of those new interventions that are effective and appropriate for everyday use in the NHS, and the process of their implementation into routine clinical practice. In clinical trials, the eligibility of patients is rigorously predetermined and experimental conditions are carefully controlled. However, once shown to be effective in a clinical trial, evidence is also required to establish the benefit of using an intervention amongst the broader patient population in routine clinical practice. Given the finite resources available to the NHS and, indeed, all health systems, data is also required to evaluate the clinical- and cost-effectiveness of new and pre-existing interventions. Moreover, research into the organisational structures that deliver those interventions can highlight where improvements and efficiencies might be made. All of the above processes, or, indeed, deficiencies in these processes, can themselves generate a gap in the translation of new medical interventions into everyday practice, in what is referred to here as the 'Second Gap in Translation.' In this context, Knowledge Management, from research observation to routine clinical practice, can be broken down into four discrete activities: Knowledge Production, Knowledge Transfer, Knowledge Reception and Knowledge Use.

Chart 7.1: Pathway for Translation of Health Research into Healthcare Improvement

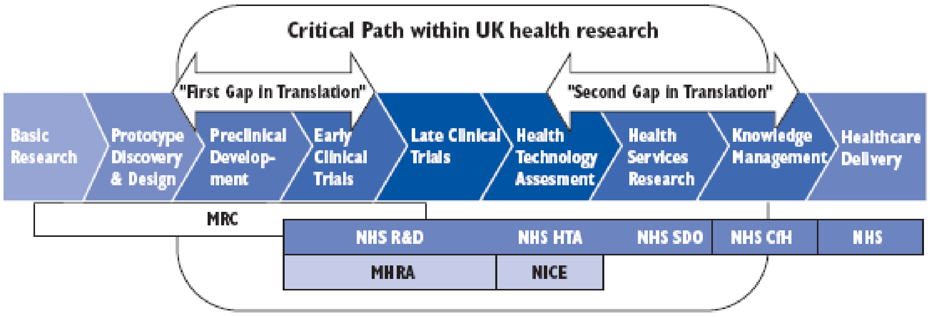

Blue boxes below parts of pathway correspond to specific responsibilities of public sector bodies supporting research. MRC: Medical Research Council. NHS R&D: National Health Service Research and Development. NHS HTA: NHS Health Technology Assessment programme. NHS SDO: Service and Delivery Organisation research programme. NHS CfH: Connecting for Health. Light blue boxes below parts of pathway correspond to the specific responsibilities of statutory regulatory agencies. MHRA: Medicines and Healthcare products Regulatory Agency. NICE: National Institute for Health and Clinical Excellence.

Addressing the Second Gap in Translation

7.56 Since its inception in 1993, the NHS HTA programme has been extremely successful in its role of Knowledge Production by providing NHS decision-makers with a

high quality evidence base, in meeting needs created by ‘R&D market failure’ and for its innovation and flexibility. Under the new institutional arrangements proposed in this report, the HTA programme should benefit significantly from a greater proportion of support for clinical research, quality and safety research and public health intervention research within the overall portfolio of UK health research. There is a crucial need for improved Knowledge Transfer, Reception and Use of HTA findings through the NHS. A substantial proportion of the escalating information needs of the NHS could specifically be met by expanding the HTA programme to:

- strengthen the commissioned workstreams for primary research, clinical trials and themed call programmes;

- generate a more systematic approach to the evaluation of diagnostic tests;

- follow up on research recommendations from NICE;

- improve the assessment of medical devices, in collaboration with the NHS Purchasing and Supply Agency;

- improve Knowledge Transfer via joint developments with the National Knowledge Service of Connecting for Health; and

- augment HTA clinical trials infrastructure.

7.57 The Review believes that this agenda will substantively increase the effectiveness, and lead to more effective use, of the NHS HTA Programme. To enhance the evidence base informing decisions on the effectiveness and cost-effectiveness of technologies in the NHS, the Review therefore recommends an expansion of the NHS HTA programme to fund these developments, which, for a relatively modest investment, could deliver large improvements in the quality and efficiency of healthcare in the NHS, and will be crucial to delivery of the Wanless ‘fully engaged’ scenario. To inform future spending decisions and to ensure that this extra investment achieves the intended outcomes, it will be important from the outset to develop a system of metrics that can accurately evaluate the impact of this expansion of the HTA programme.

7.58 With the increasing recognition of its importance and continued success, the NHS SDO Programme budget has expanded steadily, reflecting the growth in the amount of research commissioned, from £167,000 in 2000–01, to £3.7 million in 2002–03 and £7 million in 2006–07. There are plans to substantially increase the SDO programme research funding over the next three years and to allocate additional funding to new research areas, such as public health research. These plans to enlarge the programme should be developed in parallel with those for further expansion in the capacity of the NHS for Knowledge Transfer, Reception and Use. The National Programme for Information Technology, operated by NHS Connecting for Health, may be an important first step in the evolution of decision-based software systems that healthcare professionals will need in order to manage effectively the growing scale of our knowledge base and thus improve the care of patients. However, this programme is not likely to be operational at such a level for some time. Nor has it been designed specifically for the purpose of Knowledge Transfer. In any event, the uptake of new knowledge, particularly complex information, often depends the direct communication of new potential users with those familiar with that new technology, so that solutions

involving Information Technology are unlikely to be entirely sufficient. Likewise, direct marketing or information campaigns are unlikely to adequately support the spread of these types of knowledge within the health services.

7.59 **The Review recommends the establishment of a pilot programme, under the joint auspices of the NHS SDO programme and the NHS' Connecting for Health 'National Knowledge Service', to examine the effectiveness of employing a small number of full-time 'Knowledge Transfer Champions' to disseminate the findings of health services research and facilitate early adoption of those findings into routine practice in the NHS.** It will be important to develop an appropriate system of metrics from the outset to accurately assess the effectiveness of this pilot programme. Should it prove successful, the programme could be extended to include wider knowledge transfer functions within the NHS, such as the dissemination and implementation of NICE guidelines.

7.60 NICE sits at the interface between health technology assessment and clinical practice. As part of its process to develop guidance, NICE routinely identifies priorities for research that reflect important gaps in the evidence base. NICE increasingly reviews technologies closer to the stage when they will be granted a marketing license. At this stage of development, there is often only preliminary evidence on costs or effectiveness, particularly for specific disease indications and subgroups. In these cases, NICE can make recommendations for an intervention to be used "in the context of research," so that the necessary evidence can be generated to inform a future decision on its use. In other words, as a body concerned with Knowledge Transfer, it also has a pivotal role in identifying future needs in Knowledge Production.

7.61 Moreover, as part of its recently expanded remit, NICE has also issued recommendations for the use of an intervention in the context of Public Health research. For example, in recent guidance on interventions to encourage physical activity, NICE recommended the use of pedometers and exercise referral schemes only in the context of well designed trials. The guidance describes the type of research needed to assess these interventions that already take place within the NHS and the broader public sector. NICE has also, on occasion, recommended the use of an intervention in conjunction with prospective collection of information on side-effects, efficacy or costs. Such recommendations can propose the use of existing registers or clinical databases or, alternatively, the establishment of a new register or clinical database. In order to take full advantage of these NICE recommendations, it would be useful to identify resources to support this type of research and establish formal arrangements between NICE, the NHS and the commercial sector, so that the output of this research could be fed more systematically back into the NICE review process and inform future NICE recommendations.

7.62 In addition, the delivery of robust scientific appraisal for new technologies is coming under increasing challenge as a result of its reliance on methodologies that, it is widely recognised, need further development, given that HTA is a relatively new science. Appropriate research is required to address these challenges. In particular, research into methodology for:

- biostatistics;

- indirect comparisons between treatment options;

- economic evaluation of public health;

- appraisal of evidence derived from clinical trials;

- disinvestment methods;

- behavioural research; and

- the assessment of the impact of NICE guidance

should strengthen the ability of NICE to deliver its remit in the future. The Review recommends that funding be identified and formal arrangements be established between the NHS HTA Programme, NHS SDO Programme and NICE in order to:

- Implement NICE recommendations calling on the NHS to use health interventions in a research context; and

- Investigate improvements in methodologies for use in both NICE appraisals and assessments of the impact of NICE guidance on the NHS.

7.63 It will be important from the outset to develop a system of metrics that can accurately access the impact of this research in the implementation of NICE guidance.
